# Supplementary material for: Integrated Proteomics and Metabolomics Analysis of Nitrogen System Regulation on Soybean Plant Nodulation and Nitrogen Fixation
Source: Int J Mol Sci. 2022 Feb 25;23(5):2545. doi: 10.3390/ijms23052545 (PMC8910638; doi:10.3390/ijms23052545)
Supplement: Supplementary file 1 [file ijms-23-02545-s001.zip › supplementary materials/Figure S1. Preparation of plant materials with a dual-root system and nutrient solution preparation method.pdf]

Preparation of plant materials with a dual-root system and nutrient solution preparation method

Specimens were cultivated and raised in sand pots. The diameter of each plastic bucket was 0.30 m, and the height was 0.28 m. The plastic bucket was divided by a customized polycarbonate plastic board that fit the inside the bucket into two equal parts to allow two roots to grow separately. The height of the plastic board was slightly lower than the edge of the bucket at 2 cm. The gap between the barrel and the board was sealed with glue, and 1-cm-diameter drain holes were drilled at the bottom of the barrel. The overall sand content was 20 kg. The sand was washed with tap water before filling the bucket and then rinsed twice with distilled water.

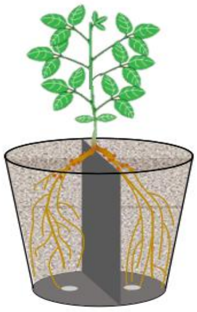

Pot plot of dual-root soybeans

Dual Root System：

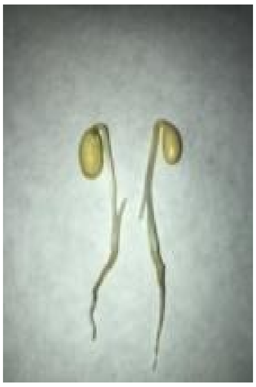

Seeds were seeded into fine-sand medium and were cultured in a growth chamber at 30° C for approximately 3 days.Cut long slightly,the nodulatied soybean had an upward incision,the non-nodulated soybean plants had a downward incision.

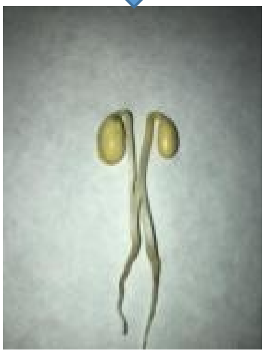

Two seedlings were cross-inserted into the cuts of each other.

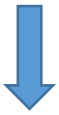

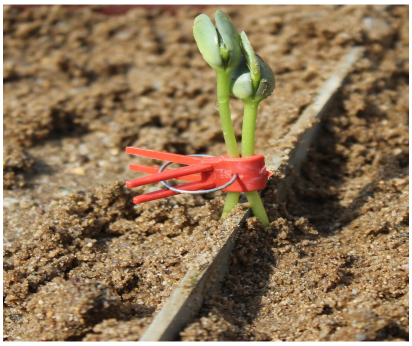

Two seedlings were separately planted into fine-sand medium of each half of the pot divided by the partition plate.

The seedlings were treated with 250 mL of distilled water on each side once a day before the opposite true leaves were fully expanded; 250 mL of nutrient solution was administered on both sides once a day after the opposite true leaves were expanded and then once in the morning and evening after the flowering period. When the true soybean leaves were completely unfolded, the field soybean nodules cryopreserved in the previous year were ground and added to the nutrient solution, with approximately 5 g of nodules per liter, and inoculated for 5 consecutive days. The nutrient composition and concentration (mg/L) of the nitrogen-free nutrient solution were as follows: KH_2PO_4, 136.00; $MgSO_4$, 240.00; $CaCl_2$, 220.00; $Na_2MoO_4{\cdot}H_2O$, 0.03; $CuSO_4{\cdot}5H_2O$, 0.08; $ZnSO_4{\cdot}7H_2O$, 0.22; $MnCl_2{\cdot}4H_2O$, 4.90; H_3BO_3, 2.86; $FeSO_4{\cdot}7H_2O$, 5.57; and Na_2EDTA, 7.45; nitrogen-containing nutrient solution was added to the experimental nitrogen source on this basis.
