# Supplementary material for: Integrated Proteomics and Metabolomics Analysis of Nitrogen System Regulation on Soybean Plant Nodulation and Nitrogen Fixation
Source: Int J Mol Sci. 2022 Feb 25;23(5):2545. doi: 10.3390/ijms23052545 (PMC8910638; doi:10.3390/ijms23052545)
Supplement: Supplementary file 1 [file ijms-23-02545-s001.zip › supplementary materials/Figure S2. Visualization of NO production in root nodules after the supply nitrogen.pdf]

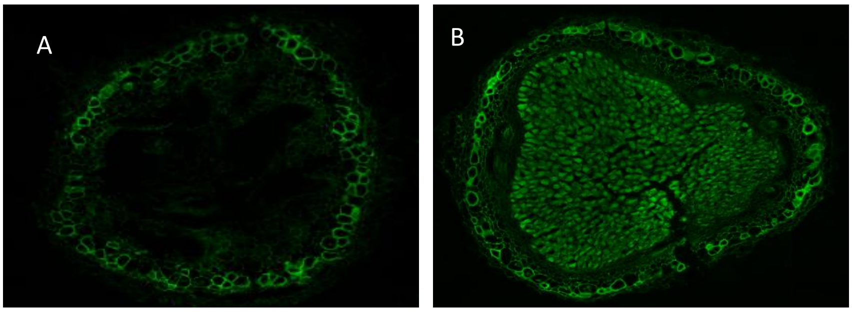

Figure S2. Visualization of NO production in root nodules after the supply nitrogen. NO production was visualized using the NO-reactive fluorescent probe DAF-FM-DA. (A) was NF treatments nodules, (B) was NH treatments nodules.
