# Supplementary material for: Integrated Proteomics and Metabolomics Analysis of Nitrogen System Regulation on Soybean Plant Nodulation and Nitrogen Fixation
Source: Int J Mol Sci. 2022 Feb 25;23(5):2545. doi: 10.3390/ijms23052545 (PMC8910638; doi:10.3390/ijms23052545)
Supplement: Supplementary file 1 [file ijms-23-02545-s001.zip › supplementary materials/Table S4. NO content in double soybean nodules after nitrogen supply.pdf]

Table S4. NO content in double soybean nodules after nitrogen supply

Treatments	NO content (μmol/g)
**NF**	0.54±0.03b
**NH**	1.45±0.06a

The data are represented as the mean values ± standard error and independent measurements with four replicates. Different lowercase letters indicate a significant difference between the treatments at the 5% level.
